# Towards the identification of Idiopathic Parkinson’s Disease from the speech. New articulatory kinetic biomarkers

**DOI:** 10.1371/journal.pone.0189583

**Published:** 2017-12-14

**Authors:** J. I. Godino-Llorente, S. Shattuck-Hufnagel, J. Y. Choi, L. Moro-Velázquez, J. A. Gómez-García

**Affiliations:** 1 Speech Communication Group, Research Laboratory of Electronics, Massachusetts Institute of Technology, Cambridge, Massachusetts, United States of America; 2 Centre for Biomedical Technology, Universidad Politécnica de Madrid, Madrid, Spain; University of Zurich, SWITZERLAND

## Abstract

Although a large amount of acoustic indicators have already been proposed in the literature to evaluate the hypokinetic dysarthria of people with Parkinson’s Disease, the goal of this work is to identify and interpret new reliable and complementary articulatory biomarkers that could be applied to predict/evaluate Parkinson’s Disease from a diadochokinetic test, contributing to the possibility of a further multidimensional analysis of the speech of parkinsonian patients. The new biomarkers proposed are based on the kinetic behaviour of the envelope trace, which is directly linked with the articulatory dysfunctions introduced by the disease since the early stages. The interest of these new articulatory indicators stands on their easiness of identification and interpretation, and their potential to be translated into computer based automatic methods to screen the disease from the speech. Throughout this paper, the accuracy provided by these acoustic kinetic biomarkers is compared with the one obtained with a baseline system based on speaker identification techniques. Results show accuracies around 85% that are in line with those obtained with the complex state of the art speaker recognition techniques, but with an easier physical interpretation, which open the possibility to be transferred to a clinical setting.

## Introduction

Dysarthria is defined as "*a group of related speech disorders that are due to disturbances in muscular control of the speech mechanism resulting from impairment of any of the basic motor processes involved in the execution of speech*" [1]. Thus dysarthria makes difficult verbal output due to lack of coordination, paralysis, or weakness of the speech musculature. Dysarthria is caused by damage in the central or peripheral nervous system. This damage may be present since birth, as in the cases of muscular dystrophy or cerebral palsy, or may take place later in life caused by one of the different conditions that can affect the nervous system, including multiple sclerosis, Parkinson, Huntington’s disease, brain tumours, stroke, injury, etc.

The perceptual characteristics of dysarthria were first studied by Darey et al. [2][3] in their seminal works. All of the 212 patients studied had signs of dysarthria in connection with different neurologic impairments (i.e., amyotrophic lateral sclerosis, bulbar palsy, pseudobulbar palsy, parkinsonism, dystonia, cerebellar ataxia and chorea). The authors identified 38 distinct speech characteristics that were categorized into seven modalities: pitch, loudness, vocal quality, prosody, articulation, resonance, and respiration. The study observed characteristics that were specific to each neurogenic group, as well as characteristics shared by more than one. After perceptual characteristics were identified and clustered to each neurogenic group, dysarthric speech characteristics were further recategorized in the following dysarthria types: ataxic, spastic, hypokinetic, hyperkinetic, unilateral upper motor neuron, flaccid and mixed.

Among the different types of dysarthria, hypokinetic dysarthria is mainly associated with dysfunctions of the basal ganglia control circuits, which are the loops that mediate between cognitive functions (provided from the Wernike’s and Brocca’s cortical areas that process the language) and motoric functions at the hippocampus. The basal ganglia regulates muscle tone, providing support for voluntary motor movements, controlling postural stability during fine motor actions, adjusting movements to the sensory status of the periphery, and assisting in motor learning [4]. The basal ganglia also smooths and refines intended motor movements. When the motor movements have been refined, the basal ganglia sends the clarified motor’s program up to the motor cortex, by way of the thalamus, where it is then forwarded through the direct activation pathway and out to the muscles [5]. If this circuit is damaged, motor movement may be excessively reduced/damped, or unwanted involuntary motor movement may not be inhibited.

There are several aetiologies of hypokinetic dysarthria. Idiopathic Parkinson’s Disease (PD) is the prototypical and most common disease associated with this kind of dysarthria. The estimated prevalence rate of PD for people aged over 65 years is 1.5% which makes this disease the second most frequent neurodegenerative disorder [6]. The low levels of dopamine that appear in patients with PD lead to dysfunctions of the basal ganglia, manifesting motoric symptoms such as tremor, reduced range of motion due to rigidity, postural instability, bradikinesia, or even akinesia, adversely affecting those tasks requiring a fine control of muscles. These deficits negatively affect the three main anatomic subsystems involved in the speech production: respiration, phonation and articulation, which are controlled by the speech motor control. In more advanced stages the disease also manifests other non-motor symptoms such as neurobehavioral and cognitive abnormality, sensory disruptions and sleep alterations. Statistics show that approximately 75–90% of PD patients develop dysarthria during the course of the disease, but the most evident signs of dysarthria are present in 60% or more people with PD, with increased prevalence as the disease progress [4][7][8][9].

**Fig 1:**
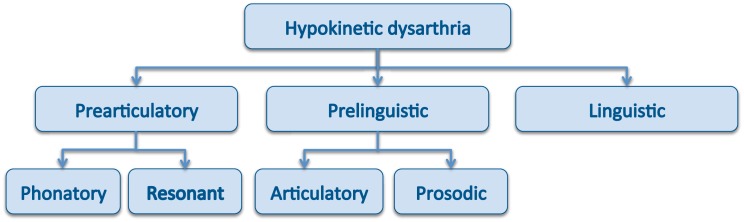
Categorization of the disturbances associated to the hypokinetic dysarthria of PD patents.

The motoric limitations introduced by PD affects the speech production process, leading to a large amount of disturbances, that roughly speaking could be categorized into three main dimensions (Fig 1): *prearticulatory* (phonatory and resonant characteristics), *prelinguistic* (articulatory changes and prosodic fluctuations), and *linguistic*. In this context, the prearticulatory dimension refers to the sound production from the physical perspective, but having in mind only aspects related with the phonation process at the level of the excitation, and the modulations introduced by the vocal tract as a time-invariant resonant structure. The prelinguistic dimension refers to changes in the prosody of the speech, reflected as intensity and f0 variations, as well as to articulatory changes, which emerge due to distortions in the speed of change, position, and coordination of the articulators, and as a result of their interaction with the excitation. Thus it refers to the speech production mechanisms as time-variant structures. Lastly, the linguistic dimension is related with changes in the expected acoustic content of the speech, mainly due to repetitions and/or substitutions. In summary, the prearticulatory and the prelinguistic dimensions are related with the speech, and the linguistic dimension with the expressive capabilities of the language.

The following sections present a review of some of the characteristics identified in the speech of PD patients associated to these three dimensions, which for categorization purposes can be split into five different levels (Fig 1): phonatory, resonant, articulatory, prosodic and linguistic.

### Phonatory

Phonatory features characterize the speech at the prearticulatory level, and were developed mainly to characterize the sustained phonation of vowels.

The acoustic analysis carried out in [10] using sustained vowels reported that PD patients present significant differences in glottal noise, and tremor. However, the tremor in the speech of PD patients is not pervasively present, being also relatively slow (3 to 7 Hz), and thus difficult to identify perceptually. Using videoendoscopic techniques, the work carried out in [11] reports that more than 55% of the PD patients analysed presented horizontal or vertical tremor in the vocal folds. The same study also reports cases of incomplete vocal folds closure along with folds bowing during phonation, leading to the presence of noise, that is typically characterized using measures such as Noise to Harmonics Ratio (NHR), Glottal to Noise Ratio (GNE), Harmonics to Noise Ratio (HNR) and Voice turbulence Index (VTI) among others [12]. On the other hand, there are works that have reported an increase of the average f0, jitter [13] and shimmer values [14], although the literature reports a certain controversy about their usefulness [15][16] to characterize the speech of PD patients. Cepstral Peak Prominence (CPP) also demonstrated to be a good quality measure to characterize PD populations [17][16]. Measures of intensity [14][18] have also reported a reduced volume, but their reliability is limited to characterize sustained vowels, since these measures are quite sensitive to the calibration of the system. Complexity measures have also been used to characterize the speech of PD patients: Largest Lyapunov Exponent (LLE), Correlation Dimension (CD), Detrended Fluctuation analysis (DFE), Hurst Exponent (HE) and Approximate Entropy (AE) among others [12][19]. Moreover, PD patients show a decreased voice range profile or phonetogram [7][20].

On spite of the interest of these features to objectively characterize parkinsonian voices, an accurate estimation of them usually requires long audio recordings of sustained phonations (i.e. more than 3 sec. long), and/or a fine calibration of the recording system. Thus, their application is quite limited when analysing continuous speech, which inherently contains short voiced segments and large variations in amplitude due to intensity modulations. On the other hand, phonatory features face a number of inherent limitations, such as characterizing spirantization, imprecise articulation, low frequency frications, voice onset time variability, etc.

### Resonant

As the previous set of characteristics, resonant features characterize the speech at the prearticulatory level using the sustained phonation of vowels.

The distortions that appear in the resonant characteristics can be explained due to an inaccurate position of the articulators or due to a certain lack of control of the velopharyngeal port.

The quality, intelligibility of a vowel, and the precision of the articulation can be determined primarily by the distinctive acoustic energy peak of the two first *formants*, the F1 and F2 frequencies. The F1 and F2 frequencies reflect tongue position, with an acoustic-articulatory relationship defined such that the F1 frequency varies inversely with the height of the tongue while the F2 frequency varies directly with its advancement [17].

In this sense, the work [13] suggests that PD patients present an asymmetric centralization of unrounded vowels (/a/, /e/, /i/) in high/low/front/back positions of the tongue during the phonation of sustained vowels, consequently leading to a notable decrease in the Vowel Space Area (VSA) in comparison with healthy controls. However, there is a certain controversy and other findings suggest that sustained phonation is not an appropriate task to study early PD [21].

Hypernasality may also occur in PD due to certain incompetences of the velopharyngeal port, including the degree and velocity of velar movements. Although its presence is not usually perceived by human experts in more than 30% of the speakers with PD, acoustic and aerodynamic studies suggest increased nasal airflow in the 70% of persons with the disease [22][23].

As the phonatory features, an accurate estimation of the resonant characteristics requires long registers of sustained phonation, limiting the possibility to use them for the analysis of continuous speech.

As with phonatory features, resonant characteristics imply a large amount of inherent limitations, which are in part addressed by the prelinguistic characteristics (i.e. articulatory and prosodic) found in the state of the art.

### Articulatory

The study of articulatory deficits requires the analysis of connected speech into components such as phonemes, words or sentences. The deficits of articulation are manifested as reduced amplitude, precision, velocity and variability of the articulatory movements of lips, tongue and jaw, leading to imprecise consonant or vowel production.

VSA has also been studied for vowels that were extracted from connected speech, thus in an acoustic context which affects the placement of the articulators. Using vowels extracted from read sentences, authors in [24] have also reported that individuals with PD have a significantly smaller VSA in comparison with control speakers. Conversely, other studies have reported no statistically relevant differences in this same measure between control and PD speakers [25][26]. On the other hand, the Vowel Articulation Index (VAI) is significantly reduced in male and female PD patients as compared with the control group [21], allowing discrimination with an accuracy around 80%, although no correlations were seen between VAI and the stage of the disease. Formant Centralization ratio (FCR) is another measure that has demonstrated differences between PD individuals and control speakers [27]. In addition, the Articulatory–acoustic Vowel Space is a measure that has shown to be sensitive to within-person fluctuations in articulatory function and disease-related group differences of PD patients [28].

On the other hand, one acoustic measure with a relatively straightforward articulatory interpretation is the slope of F2 transitions extracted from diphthongs or vocalic nuclei requiring relatively large changes in vocal tract configuration. The literature reports that PD patients have shallower F1 [29] and F2 [30][29][29][30][31] slopes compared to the control groups, indicating that the former have decreased tongue/jaw excursions. On this account, shallower slopes would be associated with a reduced velocity in the articulators and less defined spectra for stops and tors, which lead to a reduced intelligibility, in part because of weakly defined obstruent spectra [32].

In addition, [33] demonstrates that the Relative Fundamental Frequency (RFF), measured in the vowel onset and offset segments before a voiceless consonant as the instantaneous f0 of each cycle normalized by the f0 in the vowel segment, is statistically significantly lower in individuals with PD compared with healthy age-matched controls.

The literature also reports incomplete stop consonant occlusions [34]. Authors in [35] suggest that the imprecisions of articulation on stop consonants often results in spirantization or low frequency frication noise replacing stop gaps as a consequence of a reduced closure [35]. In this sense, Voice Onset Time (VOT) variability for voiced and voiceless stops has also been identified as an indicator of the presence of the disease [36][36][37][35][29].

### Prosodic

The prosodic characteristics are directly linked with the analysis of continuous speech. Disturbances in prosody frequently appear in connected speech tasks that necessitate loudness and pitch variation, such as expressing emotions, reading aloud, and in conversational speech.

Typically the speech of PD patients presents a reduced f0 variability (monopitch) [37][38][15], reduced stress, reduced rate of speech [37][34][39][40], modified syllable rate [35][34] and reduced loudness variability (monoloudness) [41]. Although less frequent, other prosodic characteristics are increased rate of the speech [39] and inappropriate silences [2][3]. The bradykinesia (reduced speed of muscles) and freezing of movement that accompanies the PD speakers sometimes causes difficulty in the initiation of voluntary speech, as well as inappropriate long silences. In contrast, with the advancement of the disease, festinating speech is sometimes observed, resulting in short rushes of speech together with fast locutions. However, most of the times, the presence of festinating speech is just a perception but not an objective fact, since the rate rarely is over the one of normophonic speakers [42][43]. It has been justified by a voluntary increase of the rate of speech to compensate the slow speech caused by the disease. Increasing the speed lets the speaker reach the speech rate of normophonic speakers but causes blurring of articulation, similar to the one that would appear in the general population speaking fast.

### Linguistic

The linguistic disturbances that appear in PD patients are mainly related with changes in the informative content of the speech (i.e. the expressive dimension of the speech), and are manifested as repetitions and/or substitutions. These distortions are caused by changes linked to the progression of PD, since they are more prevalent in late stages of PD, although some works suggest changes from the beginning [44]. The prevalence of these disturbances is no more than 30% of the PD population [45].

There are two variants of speech repetitions, one hyperfluent, known as pallilalia, and another dysfluent, suttering-like. Pallilalia [46], or the compulsive repetition of words, or even phrases, is sometimes present in PD patients, but no more than in 15% [45] of the PD population. Due to the poor articulation and decreasing loudness, these repetitions become blurred. However, stuttering-like repetitions are usually relatively well articulated at a constant rate and loudness, and their prevalence is similar to the former one. Although the role of repetitions of speech in PD is not well known, they are commonly linked to a deficit of the motor speech control, associated to the freezing symptom, but cognitive and linguistic factors (at the prearticulatory level) seems to contribute to their generation [45].

On the other hand, the authors of [47] have found that the number of dubitative silences and their duration is more significant in PD patients than in controls, as well as the number of interjections. Some works have identified problems to enumerate categories in tasks that are not associated to the memory and have linked them with the degree of bradikinesia [48]. There are also studies that present substantial differences between PD and control groups in their linguistic abilities while answering certain questions [49] and in the use of formulaic expressions [50].

### Problem statement

In summary, the reduced range of movement, decreased precision, tremor, and muscle rigidity are the most significant neurogenic symptoms associated with the hypokinetic dysarthria of PD patients [4], limiting the required ability to produce the rapid and accurate movements needed to generate the speech [5] and leading to what many works have described as an articulatory undershoot. As already seen, the literature has proposed a large amount of measurements, with each feature leading to different sensitivity and specificity values depending on its prevalence in the disease. Despite of the fact that some of these features have demonstrated to be strongly correlated with the presence of PD, their translation to automatic computer-based screening/evaluation systems is not straightforward. In addition, these three dimensions cannot be easily combined in a single computer-based system, since they require different methods and/or acoustic materials, increasing the complexity of the detector. To this respect, in the last few years, the literature reports computer-based multidimensional approaches to evaluate the hypokinetic dysarthria, although they are not integrated yet in the clinical setting due to their complexity and difficult physical interpretation. As a matter of example, two multidimensional analysis, at the prearticulatory and prelinguistic dimensions, are presented in [12] and [51] respectively.

Despite of the evidences of correlation between PD and the speech quality measures identified in the literature, the lack of easy to interpret objective indicators capable of identifying PD with a desirable level of sensitivity and/or specificity, and transferable to computer-based systems, has limited the role of the speech analysis in the overall evaluation process of PD. Consequently, the most common protocols used to evaluate the extent of PD in the clinical practice, such as UPDRS-III [52], take the speech into account only at an advanced stage of the disease and based only on perceptual judgements. Despite of the fact that the perceptual analysis is still used as a gold standard, it has been criticized in the literature due to the required training and its inter and intra evaluator variability [53]. Besides, certain symptoms may be affected by other disturbances (e.g., the rate of speech may affect the ability of clinicians to accurately judge articulation), leading to a certain confusion.

Thus there is a need of new robust biomarkers, especially at the paralingustic-articulatory level, which could be objectively translated into the clinical practice to objectively document the speech of PD patients, and to be used for longitudinal studies. These indicators are expected to be extracted in an automatic manner to further be transferable to computer based systems, replacing the perceptual judgements with more objective methods not biased by the experience of the evaluator.

To this end, this paper proposes new articulatory biomarkers based on the kinetic behaviour of the envelope trace, which is supposed to be directly related with the articulatory dysfunctions introduced by the disease since the early stage. A performance comparison is also carried out with a baseline system based on speaker identification techniques.

The paper is organized as follows: section 2 presents a review of the acoustic material used and the corpus of speakers, a collection of examples to present the type of disturbances that usually appear in parkinsonian speech and the methods used to obtain both the acoustic biomarkers and the baseline systems used for comparison purposes; section 3 presents the results obtained with the baseline systems and with the methods proposed; and section 4 is dedicated to the discussion and conclusions.

## Methods

The evaluation carried out throughout this work is based on the acoustic material extracted from segments of repeated speech. More precisely from a diadochokinetic (DDK) test. This section provides a review of the DDK test, as well as of the database of speakers used. Besides, the characteristics of the speech in normophonic speakers during the aforementioned test are presented to fix the basis of the deviations seen in the speech of PD patients.

Moreover, throughout the study of some cases, this section describes some of the evidences of the speech of PD patients that have been used as a basis to develop the proposed acoustic biomarkers.

### The diadochokinetic test (DDK)

The DDK task [54] is a clinical test employed in the assessment of the functional capacities of the articulatory system. The rationale to use this test stands on the fact that motor deficits in the speech capabilities of PD individuals manifest themselves more strongly under circumstances requiring motor planning and execution over extended sequences of motor production (i.e., when a single sequence is repeated several times or cases in which a given fragment occurs within a sentence context). Compensation for PD results in a simplification of the articulation at the expense of ease of processing [55], revealing important cues about speech production from the motoric point of view, even when the DDK task is simpler in motor terms than the speech taken from more natural language data (i.e. conversational speech).

The test involves the alternate production of syllable sequences requiring a fine articulatory precision, together with the capability to rapidly change articulators between two consecutive segments. This is typically carried out asking the patient to produce combinations containing vowels and voiceless consonant with bilabial, alveolar, and velar places of articulation. Specifically, the subjects are typically asked to repeat the sequence of syllables /pa/-/ta/-/ka/ unceasingly for about 10 seconds as clear and as fast as they possibly could. This test requires rapid movements of the articulators, using the lips (the front), the tip of the tongue (middle), and the soft palate (back of the mouth), sequentially and continuously.

Despite of its simplicity, this task reveals some cues about the speaker’s ability to produce the speech with an adequate rate, and to evaluate syllable-to-syllable stability or subphonemic durations [55]. In this sense, the syllable rates of the DDK test are used as an indicator to evaluate the patient’s ability to rapidly alternate speech movements [54]. On the other hand, the test is also used to assess imprecise consonant coordination measuring the VOT, typically determined as the duration between the initial burst and the vowel onset [56].

### Corpus of speakers

**Fig 2:**
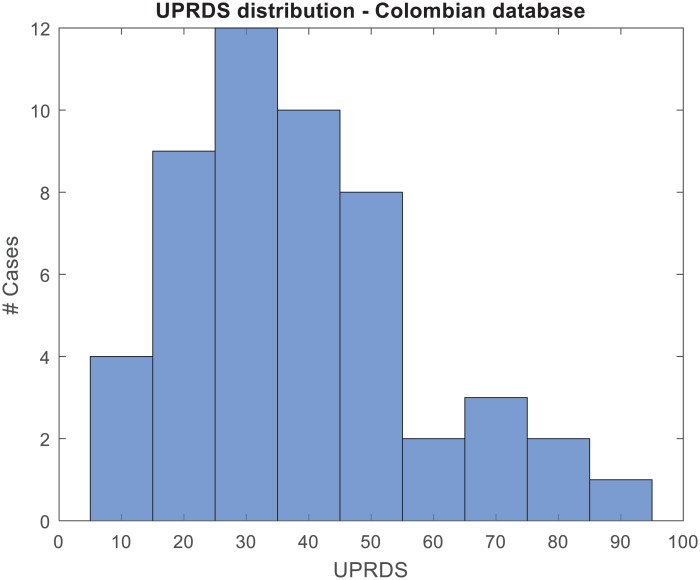
Histogram of the UPDRS-III labels of the corpus of speakers.

The speech traces used in this study were extracted from the PD speech database recorded in Medellín (Colombia) [57]. This database contains speech recordings from 50 patients with Idiopathic Parkinson’s Disease and 50 healthy speakers sampled at 44,1 kHz with 16 bits of resolution. The speakers are equally balanced by gender and balanced in age, being Spanish their mother language. The age of the parkinsonian female patients ranges from 44 to 75 (mean 60,1±7.8) and from 33 to 77 years old (mean 62,2±11,2) for males. The age of the healthy females and males ranges from 43 to 76 (mean 60,7±67,7) and from 31 to 86 (mean 61,2±11.3) respectively. The average duration of the disease prior to recording was 10,7±9,2 years. All the patients were diagnosed and labelled according to the UPDRS-III [52] and Hoehn & Yahr [58] scales by neurologists, with mean values of 36,7±18,7 (Fig 2) and 2,3±0,8, respectively. The speech samples were recorded no more than 3 hours after the patient’s morning medication (i.e. in ON-state). None of the people in the control group had a history of symptoms related to PD or any other kind of neurological disorders. The database contains a large amount of acoustic material including words, vowels, sentences, reading text, DDK tests, etc. Only the DDK recordings were used in this study.

### Characteristics of the DDK test waveform in the normophonic speaker

For the ultimate goal of comparison, first the study presents an analysis of the speech of a normophonic speaker uttering the DDK test.

**Fig 3:**
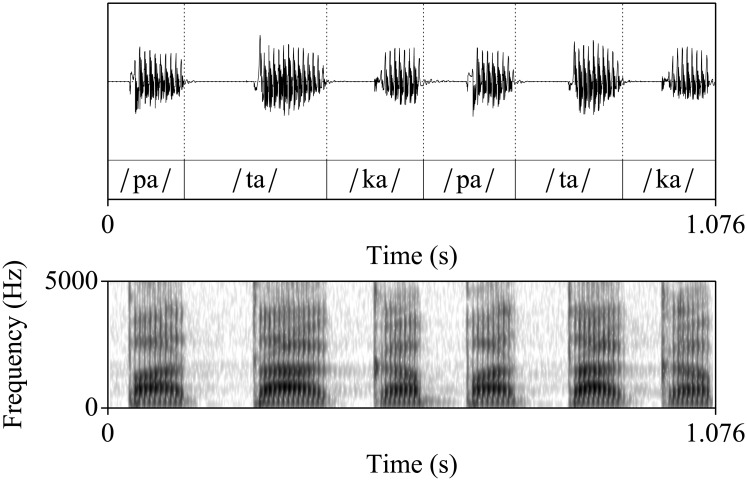
Speech waveform and spectrogram of a 35 years old normophonic speaker uttering the /pa/-/ta/-/ka/ test.

**Fig 4:**
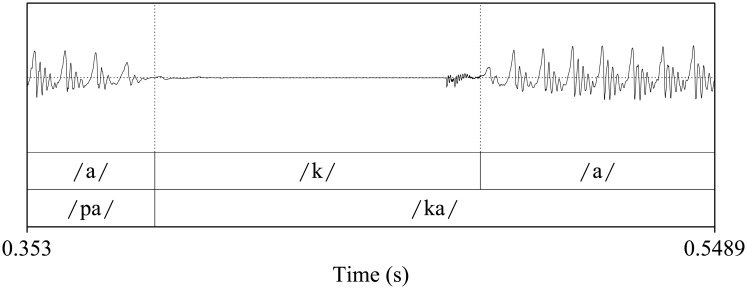
Detail of the speech corresponding to the /ka/ syllable of a 35 years old normophonic speaker. The syllable starts with a stop gap (silence), followed by a burst that is previous to the periodic sound of the vowel. The structure depicted is typical of the plosive consonant-vowel combinations used in the DDK test.

Fig 3 shows the speech trace and the spectrogram of a normal speaker uttering the /pa/-/ta/-/ka/ test. Fig 4 depicts a detailed view of the waveform presented in Fig 3 showing its time domain structure, which is common for each of the three aforementioned syllables. In the Spanish language, voiceless stops (such as /p/, /t/ and /k/) in initial position present a stop gap (silence) before the release burst. The importance of this stop gap in the perceptual identification of the different sounds is described in [59]. The phoneme corresponding to the consonant is followed by a periodic waveform corresponding to the vowel. Thus, this structure can be divided into five segments: stop bap, burst segment, vowel onset, stable part of the vowel, and vowel offset; being common to the three consonant-vowel combinations of the syllables used in the DDK test. This pattern is periodically repeated along time during the test. The time and frequency details of each of these segments are presented in Table 1.

**Table 1:** Segments in the consonant-vowel combinations of the DDK test with their corresponding time and frequency characteristics.

	Description	Frequency	Time
**Stop gap segment**	Silence before the initial burst in which the vocal tract remains closed or partially closed	Characterized by a low amplitude background noise, sometimes affected by formant frequencies.	Very low amplitude signal
**Burst release segment**	Starts with an abrupt onset of noise energy caused by turbulent airflow during the stop release at the lips, jaw or tongue.	Excitation over the entire frequency range	Short and small amplitude signal. Its duration corresponds with the Voice Onset Time (VOT).
**Vowel onset segment**	Abrupt onset of a periodic signal. The vocal folds start their vibration.	Onset of f0 and formants. Energy is concentrated in these frequencies	Signal with increasing amplitude, characterized by its periodicity
**Stable vowel segment**	Stable periodic signal. The vocal folds are vibrating.	Stable of f0 and formants. Energy is concentrated in these frequencies	High and stable amplitude, and periodic. At least requires two pitch periods of maximum amplitude.
**Vowel offset segment**	Slow voice weakening, and therefore slow weakening of f0 and formants. Corresponds with the closing of the vocal tract. Its end is difficult to identify due to weak time and energy contrast, but also due to potential non complete closure of the vocal tract.	Energy of f0 and formants decreases. The offset of the F2 is the best indicator of occlusion onset.	Decreasing amplitude. The periodicity disappears giving way to a silence.

The physiological process followed to produce the sequence of sounds of the DDK test requires the articulators to move in a fast and complex sequence able to alternate vowels and voiceless stops. The process to produce the voiced sounds is well known: roughly speaking, the sound is produced at the vocal folds that vibrate due to the air passing through them while are tightly adducted; the opening and closing (i.e. the vibration) of the vocal folds is the consequence of an attempt to compensate the gradient of pressure between the trachea and the oral cavity that recurrently appears for each cycle; the vocal tract later modulates the sound produced at the glottal level. The unvoiced stops in medial position are due to the closure produced in the vocal tract at labial, alveolar, or velar levels during the stops, so the airflow flowing through the glottis is accumulated in the oral cavity, causing oral pressure to approach subglottal pressure (i.e. at the trachea level). At the same time, the vocal folds are lightly adducted (i.e. an adduction gesture is produced). The process requires the velopharyngeal port to remain fully occluded to avoid any leak of pressure. These three mechanisms contribute to make the trans-glottal air pressure differential drop below what is required to keep the vocal folds vibrating, and the air flow diminishes, so voicing ceases, typically during 50–60 ms for an alveolar stop (slightly earlier for a velar, and later for a labial). The abrupt release of the closure later produces the burst segment (typically 20 ms long). During the production of the voiceless stops the vocal folds are appropriately adducted and tensed for voicing but with no vibration [60]. Thus over the DDK test, only the cross-sectional area of the mouth is varied, first to produce a constriction in the mouth and then to release it.

On the other hand, viewing the spectrogram of Fig 3, it is possible to extract complementary information which helps to evaluate not only the time-frequency content of the speech (i.e. f0, formants, burst segment) but also the degree of opening of the vocal tract for each speech segment. In this regard, the formants can be clearly identified in the voiced sounds, but are also present in some of the non voiced segments. As a matter of example, during the stop gap of the /ka/ syllable the first and second formants are still lightly visible, indicating an almost total but non complete closure of the vocal tract during the production of the /k/ phoneme. Besides, although less significant, this effect is lightly observed during the silences corresponding to the /ta/ syllable. However, the formants nearly disappeared during the stop gap of the /pa/ syllable, suggesting an almost complete closure of the vocal tract.

Since PD introduces a blurring effect in the speech, deviations from this pattern are expected, especially due to the articulatory and coordination deficits commented before, that would affect not only the duration of the segments identified, but also their time and frequency domain structure.

### Characteristics of the DDK test waveform in the parkinsonian speaker

**Fig 5:**
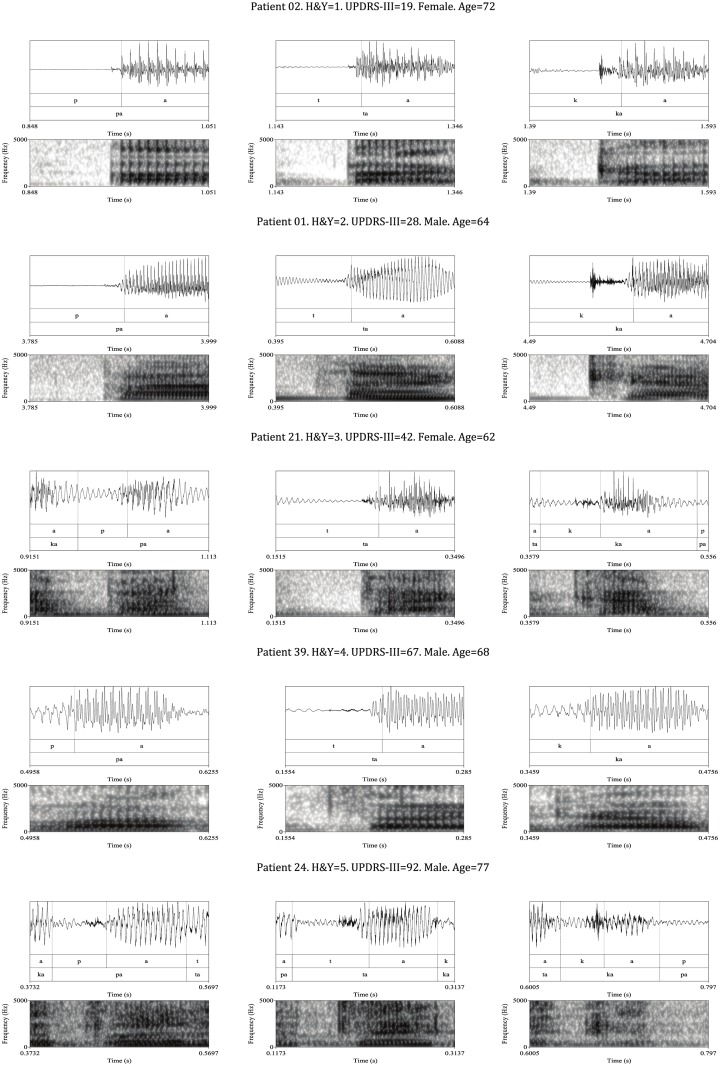
Speech trace and spectrograms of voiceless bilabial/alveolar/velar (left/center/right) stops uttered by five PD patients with different degrees of the disease according to the H&Y and UPDRS scales.

Fig 5 shows different voice traces and spectrograms corresponding to PD patients with a different extent of the disease that were recorded during the DDK task. The first column shows the acoustic environment of a voiceless bilabial stop followed by an /a/ vowel to produce the syllable /pa/ (scenario 1). The second shows the acoustic environment of a voiceless alveolar stop followed by an /a/ vowel, leading to the syllable /ta/ (scenario 2). The third column corresponds to the acoustic environment of a voiceless velar stop followed by an /a/ vowel to produce the syllable /ka/ (scenario 3). These three scenarios previously had an /a/ vowel sound, so the stops appear in a medial position. In general terms, with independence of the extension of the disease, the plots shown follow the expected circular structure for the DDK test previously commented: voiced segment, vowel offset, stop gap, burst segment, vowel onset, voiced segment, vowel offset… But a detailed view of it reveals significant differences with respect to the normophonic pattern. The following paragraphs provide some insights to analyse the speech of PD patients throughout the study of five cases of patients with a different extent of the disease according to the UPDRS-III and H&Y scales. These five examples are not presented to illustrate the whole range of disturbances that could appear in the speech of PD patients, but also to provide an insight to several deviations with respect to the normophonic pattern during a DDK test, and to justify the use of this test in the context of the present work. A more in depth analysis and justification of these disturbances is given next.

The first row of Fig 5 corresponds to a patient in the early stages of the disease that was evaluated with an H&Y rate of 1 (UPDRS-III = 19). The temporal structure of the waveforms is similar to the one corresponding to the normophonic speaker, but presents a certain ripple in the stop gaps of the three scenarios, which is more visible in the /ka/ sound. The bursts can be easily identified in both the temporal and spectrographic domains, but the amplitude of the vowel in its stable part is not constant, showing a certain degree of shimmer and/or tremor.

The second row corresponds to a patient that was evaluated with an H&Y rate of 2 (UPDRS-III = 28). In this speaker the bursts still can be identified in the temporal and spectrographic plots. As in the first example, the amplitudes of the vowels are not constant. Nonetheless, the main deviation with respect to the normophonic pattern is again a ripple in the stop gaps of the three syllables, which is more visible in the /ta/ and /ka/ sounds. A detailed analysis of this ripple reveals a fundamental frequency quite similar to the one of the vowels, but its structure is quasi-sinusoidal, meaning that even when the vocal tract is almost closed, its closure is not complete and the vocal folds are still vibrating. This is confirmed by the presence of F1 in the spectrum in absence of F2. Besides, the burst segment of the /ta/ syllable appears overlapped with the vowel sound, meaning that the vocal folds started to vibrate before the instant of closure that produces the burst, when the tongue tip touches the soft palette. This fact clearly demonstrates a lack of coordination of the articulators.

The third row corresponds to a patient that was evaluated with an H&Y rate of 3 (UPDRS-III = 42). For this speaker, the starting of the burst segment still can be identified in the temporal domain for the /ta/ and /ka/ sound, but not in the /pa/ syllable that requires an analysis of the spectrogram to see that it is overlapped with the vowel. The amplitudes of the vowel segment are also non constant and present a certain amount of noise in the /ta/ and /ka/ syllables. This noise is attributable to a closure defect of the vocal folds during the vowel production. Once again, a significant ripple appears in the stop gaps of the three syllables with a frequency similar to the f0 of the speaker. This is more visible in the /pa/ and /ka/ sounds. Note that the ripple appearing in the /ka/ syllable preserves the F1 and F2 in the spectrum, again meaning that the vocal folds are vibrating and that the vocal tract remains lightly open. Only the F1 is visible for the /pa/ and /ta/ syllables during the voice bar, meaning that the vocal tract is almost occluded.

The fourth row corresponds to a patient that was evaluated with an H&Y rate of 4 (UPDRS-III = 67). As in the previous cases, a periodic waveform is also present in the stop gaps. The burst cannot be identified in the /pa/ and /ka/ sounds, although the spectrogram for the second shows a sudden change in the spectral characteristics that could be associated with the onset of a burst segment. In fact the /pa/ and /ka/ syllables are produced by means of a modulation of the amplitude of a vowel sound, seen as a type of assimilation of the consonant to the surrounding vowels, in an example of a synchronic sonorizing lenition close to a flapping. The ripple appearing in the /pa/ and /ka/ syllables preserves the F1 and F2 in the spectrum, meaning that the vocal folds are vibrating and the vocal tract is open. The /ta/ sound only conserves the F1, meaning vibration of the vocal folds and closed vocal tract.

The last example in the fifth row corresponds to a patient that was evaluated with an H&Y rate of 5 (UPDRS-III = 92). Once again a periodic waveform is also present in the stop gaps. Surprisingly, despite of the large extend of the disease, the burst segment can still be identified in the temporal and spectrographic domain. The vowel of the /ka/ sound presents a very low amplitude and a significant amount of noise.

Voicing requires the vocal folds to be adducted (i.e. sufficient strength in the vocal folds), and sufficient gradient of pressure between the subglottal and oral tract to let the vocal folds vibrate. Both of these conditions are approached during the transition of the vowel sounds to the voiceless stops of the DDK test, so the likelihood that the stop gaps become voiced during the speech can increase due to five potential articulatory deficits. The first deficit occurs when the duration of the stop is too short (less than 40–50 ms) to allow previous voicing extinguish [60] since the shorter a stop gap is the more likely to become voicing. The second occurs when the adduction gesture of the vocal folds is not strong enough [60] which can be caused by limited glottal tension, making the vocal folds more susceptible to oscillate. The third possible cause of stop gaps becoming voiced is related to a decrease of the level of activity of the muscles which underlie the walls of the oral tract [60], actively enlarging the volume of the oral cavity therefore decreasing the pressure. The fourth and the fifth deficits are caused by an incomplete closure of the oral tract (i.e. at labial, alveolar or velar position), or if the patient has some velopharyngeal incompetence (i.e. the nasal tract is not appropriately closed), respectively. These five articulatory deficits are not mutually exclusive.

The first of these five potentialities is inherent to the formulation of the DDK test, explaining *per se* some individual cases, but prototypical durations remain in values over 40–50 ms [61], so this is not considered the main reason to explain this voicing effect. Presumably, a soft adduction gesture would lead to a lack of constriction during the voiceless stops at glottal level, and a lower level of activity of the muscles of the oral cavity leads to a relaxation of the vocal tract walls that could contribute to increase its volume, and consequently the gradient of air pressure. This, in combination with non-complete constrictions at the level of the oral or nasal tract would produce the effects observed.

**Fig 6:**
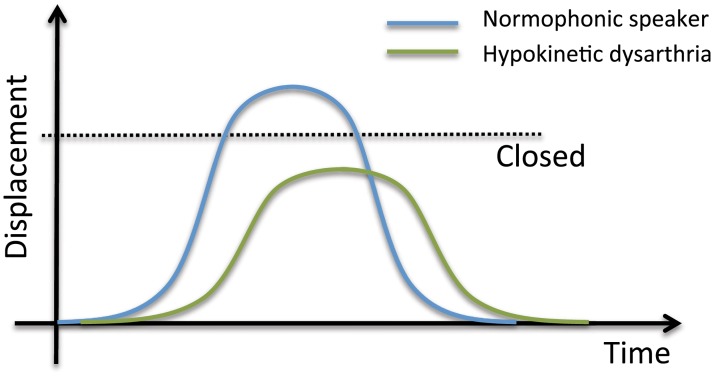
Analogy of the movements of the articulators in PD patients. Speakers do not move the articulators to their largest extent, with the required acceleration, and during the required time.

These articulatory deficits are consistent with a reduced range of movements developed by the patient to lower the “effort” or the cost needed to produce the sounds, struggling to alleviate the deficits of the motor control. Thus, this voicing process that appear in PD patients can be understood as a species of lenition, in which speakers do not move the articulators to their largest extent (i.e. closing is not complete, and adduction gesture is too soft), with the required acceleration (i.e. movements are not as fast as needed), and during the required time (i.e. movements are usually longer) (see Fig 6), in an attempt to save efforts seeking for an easy articulation or to decrease the articulation cost [60].

To this regard, [62] proposes a general effort-based theory of speech lenition that can also be applicable to the study of PD: displacement over time equals velocity; thus, to increase the displacement maintaining time invariable, velocity must increase; therefore, starting from an initial position at time *t*_a_ in which the articulator is stopped (v(*t*_a_) = 0), the greater the displacement associated to a gesture at time *t*_b_ (being *t*_b_>*t*_a_), the greater the velocity; and the greater the velocity at time *t*_b_, the greater the acceleration. Finally, as acceleration increases, the force increases (considering that mass remains constant) and consequently, the greater the effort required by the speaker to produce a sound.

This is also consistent with the fact that voiceless stops are usually considered "fortis," normally involving greater muscular force, and voiced stops, "lenis". Provided that “fortis” stops result in greater general tensing of the walls in the vocal tract, this too favours devoicing as it reduces the ability of the oral cavity to expand passively [62].

Thus, summarizing, it is possible to identify three main phonetic scenarios in which average voicing processes of PD patients during the DDK test can be viewed as the achievement of a diminution of effort cost: reduction to a briefer oral constriction at labial, alveolar or velar position; passive voicing, by elimination or reduction of the glottal adduction gesture; and not complete closure of the oral/nasal tract. Plausibly, in most cases, the lenition process of PD patients is attributable to a combination of these three scenarios.

The examples shown before confirm the lenition process during the stops towards a pattern similar to the one expected for voiced stops (/ba/-/da/-/ga/). In some extreme cases, the leak of air might be at the velopharyngeal port, in which case a nasalization process could be observed, leading to a pattern that could have a certain tendency towards a pattern like /ma/-/na/-/ŋa/.

In any case, generally speaking, and under the assumption of a constant subglottal pressure, the effects observed mean that the PD speakers maintain the phonation of the vocal folds almost during the whole exercise, primarily due to the lack of a complete closure of the vocal tract (i.e. oral and/or nasal) during the stops, but also due to a light glottal adduction gesture, and/or due to a soft tension of the muscles of the oral tract. This effect is consequent with a limitation in the displacements of the tongue, which should produce an incomplete closure of the vocal tract at velar or alveolar position, or an incomplete closure of the lips to produce voiceless stops. The flapping effect that is also observed in some of the scenarios presented is consequent with a difficulty to restore rapidly the cues for the next step of motor activation.

Another important effect is the overlapping of the vowel onset and burst segments. This reveals a lack of coordination, meaning that the vocal folds began to strongly vibrate during the release, probably due to nasalization.

On the other hand, depending on the scenario analysed, the voicing is associated to an open or almost closed vocal tract, depending on the presence or absence of F2 respectively. A close look to the behaviour of the F2 shows that in some cases it evanishes, confirming this fact.

In addition, due to the voiced ripple that appears, in some cases there is certain confusion between the stop gap and the burst segments: eventually, the burst instant is difficult to identify in the time domain, requiring an analysis in the spectrographic plot. In other cases, the burst instant simply cannot be identified.

Although not analysed in detail, subphonemic durations are also altered: VOT is reduced for PD patients [56] due to imprecise consonant coordination; and the duration of the vowel onset and vowel offset segments increases, whereas the duration of the stable vowel decreases [55], in consonance with a hysteresis effect that is linked with the inertia (i.e. the time constant) of the control system to reduce the articulatory cost of the speech production. Moreover, the syllable rates of the DDK test are also modified as an effect of the difficulties to rapidly alternate speech movements [54].

### Hypothesis

Having the aforementioned in mind, the new articulatory biomarkers identified in this work are based on the following two hypotheses:

*Hypothesis* 1: The kinetic energy of the mid-term air flow pressure (i.e. integrated at time scales comparable to the movements of the articulators) that produces the speech during the DDK test tends to vary more smoothly in PD patients than in normal control people due to the lack of closure of the tract during the stops, an inappropriate tension of the vocal folds, or due to an inaccurate control of the closure of the velopharyngeal port. In the worst scenarios, the mid-term air flow pressure tends to be almost constant with light increments in the amplitude at the vowel positions. On the other hand, there are small short/mid-term variations in the air flow pressure introduced by the muscular tremor present at the articulators, especially at the glottal level.

Having in mind that the kinetic energy is proportional to the mass and the squared velocity and under the assumption of constant mass, an analysis of the variations of the velocity of the mid-term air pressure would be of interest to study the kinetics of the speech of PD patients. This effect can be indirectly studied through the envelope of the speech, due to its relationship with the mid-term airflow pressure.

*Hypothesis* 2: The mechanism to control the articulators in patients with PD is coarser than in control subjects. In order to superimpose this, but maintaining the intelligibility as much as possible, patients produce smoother, slower and less extended movements, which for the DDK test typically lead to a lenition from voiceless to voiced obstruents. This means that the distance travelled by the articulators is less than expected, the time required to change their position is larger, and the forces involved in the control system in the mid-term are lower. This is in consonance with the idea that voiced obstruents are "lenis" and voiceless obstruents, "fortis", the latter implicating higher muscular force. Presumably, a stronger gesture eases to maintain an occlusion, but in addition requires a finer control of the articulators. In contrast, there are small short/mid-term uncontrolled variations in the displacements of the articulators due to the muscular tremor, especially at the glottal level.

Considering a constant mass, and the relationship between acceleration and force, it means that the smoothing effect reduces the forces involved to move the articulators; but the tremor introduces new forces, leading to a new scenario of changes in the effort (i.e. the distribution of forces) required to produce the speech, thus the acceleration of the articulators should present modified patterns.

Since the variations of the envelope of the speech are supposed to be indirectly linked with variations in both the mid-term air flow pressure (hypothesis 1) and/or in the distribution of forces used to control the articulators (hypothesis 2), the envelope of the speech is supposed to be affected by both hypotheses. Thus we think that the study of the envelope during a DDK test would be of interest to identify and evaluate the speech of PD speakers, so its speed and acceleration variations could provide new insights about the presence of the disease.

### Evaluation frameworks

Throughout this work, the identification of parkinsonian speech is carried out using two different approaches. The first one, which will be used as a baseline for comparison purposes, is based on state of the art speaker verifications techniques. The second proposes a new methodology to evaluate the speech of PD patients based on a kinetic approach analysing the velocity and acceleration of the envelope.

#### *Framework 1*. Speaker verification techniques

Inspired by the speaker recognition techniques we have evaluated the performance of several PD speech detectors. For the sake of comparison, results of the proposed methodologies are compared with several baseline systems developed using current state of the art techniques in text dependent automatic speaker verification based in two classification methods: GMM-UBM [63], and iVectors [64]. Both methods were used to build the statistical models of the normal and parkinsonian speakers and to evaluate the likelihood that a speech sample belongs to each class.

On the acoustic front-end the speech signal was normalized in amplitude and framed using Hanning windows with an overlapping of 50%. The potential influence of the length of these windows was tested in the range {10, 15, 20, 25, 30, 35, 40} ms. Two different short term approaches were considered to parameterize the speech signal: one based on the classic MFCC [65] and another based on RASTA-PLP [66] coefficients, both in combination with their first and second derivatives, extracted using FIR anti symmetric filters with 9 and 3 coefficients respectively. The number of MFCCs tested ranged from 10 to 20 with steps of two (i.e. {10, 12, 14, 16, 18, 20}), and the number of RASTA-PLP parameters tested ranged from 6 to 20 also with steps of two (i.e. {6, 8, 10, 12, 14, 16, 18, 20}). In the MFCCs case, for each speaker cepstral mean subtraction was carried out to normalize the feature set. The method does not consider silence removal because, as discussed in the previous section, stop gaps have demonstrated to contain very important information to evaluate PD.

For the GMM-UBM systems, the UBM was trained from a phonetically balanced subcorpus of the Albayzin Database [67] (164 speakers with gender balance), using a Maximum a Posteriori (MAP) adaptation [63] to derive the specific GMMs for both classes. Only the means were adapted, as usually done in speaker recognition [63]. The MAP approach was preferred for its ability to adjust the models even with a small number of speakers. The number of gaussians used for the GMM model was ranged in the set {4, 8, 16, 32, 64, 128, 256, 512}. In the GMM-UBM experiments, the influence of the proposed window lengths is tested. For the iVectors experiments only the length leading to best results in the GMM-UBM experiments is considered. In this latter case, the Albayzin database was also used to estimate the UBM, as well as to calculate the total variability matrix, the Linear Discriminant Analysis (LDA), the Probabilistic Linear Discriminant Analysis (PLDA), dimensionality reduction and the compensation matrices [64]. Then, two iVectors models were derived after enrolling the normal and parkinsonian speakers with the parameters and models calculated using the Albayzin database. The number of Gaussian components was also chosen in the set {4, 8, 16, 32, 64, 128, 256, 512}. The dimension of the variability space of the iVectors was ranged in the set {30, 50, 100} while the dimension of the vector of latent factor dimension varies in the range {2, 6, 10, 14, 18}.

Therefore, a large number of models are assessed, each one corresponding to a different combination of the different degrees of freedom such as type of parameterization, classification method, number of coefficients, window length (only with GMM-UBM) and other parameters related to classifiers as number of gaussians or length of iVectors.

The training was carried out using a different subset of the speakers than the one used for validation purposes. A leave-one-out technique was used to evaluate the performance of the system. These baseline schemes were developed using the Microsoft Research Identity Toolbox for Speaker Recognition [68], publicly available.

#### *Framework 2*. Kinetic approach: Evaluation of the velocity and acceleration of the envelope

The velocity and acceleration of the articulation have been indirectly estimated by parameterizing the velocity (Δ) and acceleration (ΔΔ) of the envelope of the acoustic trace during the DDK test.

The methodology of the kinetic approach is divided in two main parts. In the first stage, only one complexity measurement performed over Δ and ΔΔ, namely Permutation Entropy (PE) [69], is used to separate controls from PD patients from the database using a basic classification method.

PE has been selected as complexity feature to tune the length of the kernels since it has proven to be a useful and efficient indicator to face a number of significant problems in time series analysis, for instance: characterizing break points in time series; categorizing different dynamics; predicting future events; assessing the divergence between time series; determining time scales; or identifying causality and directionality. In that sense, PE quantifies the probability that, within a signal, a segment will resemble to the next. Equally, changes in the direction of the signal (sign of the slope), results in increases of complexity, while a steadily positive or negative slope would be associated to less complexity. Thus, a signal having only one phase per cycle would have lower complexity than other having cycles with various phases. The value of PE is represented by a unitless number ranging from 0 to 1, with lower values related to lower complexity. PE has two degrees of freedom that were adjusted according to the methods presented in the appendix.

Multiple tests are performed varying the different parameters involved in the Δ and ΔΔ calculation. The obtained results are used only to identify the optimum parameters to obtain Δ and ΔΔ of the envelope. In the second stage, a more complex detection scheme is analysed. In this case, velocity and acceleration of the envelope are calculated using the optimum parameters obtained in the previous stage to perform seven complexity measurements. The resulting features are used in a more sophisticated classification scheme to separate PD patients from controls.

In the first or preliminary stage, the envelope is automatically calculated following a procedure based on interpolating with a spline over the local maxima separated by at least 0,85·f0 s., being f0 the average fundamental frequency of the speaker estimated using [70]. Later, the envelope is downsampled to a sampling frequency of 5 kHz (from 44,1 to 5 kHz). Once the envelope is estimated, its Δ is calculated using a FIR anti-symmetric filter with a kernel based on the derivative of a gaussian with variance equal to 0.335 times the length of the filter. The ΔΔ was calculated following a similar procedure than the one for the speed. Both, Δ and ΔΔ were normalized in the [0, 1] range. Due to the smoothing effect introduced by the kernel, the selection of its length is a trade-off to avoid a noisy estimation and to precisely capture the articulatory movements. Since the median duration of an phoneme is typically in the range 70–90 ms [71], this is hypothetically the limit for their durations to avoid an oversmoothing effect. In this work, the length of the kernels have been fixed empirically to minimize the Equal Error Rate (EER) as a figure of merit to separate normal and parkinsonian speech from the Δ and ΔΔ sequences synthesized using a training subset of the corpus of speakers. The method is based on calculating the permutation entropy (PE) [71] in fixed-length speech frames of 1.37 s, overlapped an 80% and ensuring that each one contains at least 3 repetitions of the /pa/-/ta/-/ka/ sequence. The larger the duration of the frame, the better the posterior estimation of the parameters would be. However this value was fixed to 1.37 s for practical purposes, since this is the maximum duration of one of the registers available in the database.

Results at this stage were obtained following a cross validation procedure that used a k-folds method with 11 different folds randomly extracted from the database, and averaging the results.

**Fig 7:**
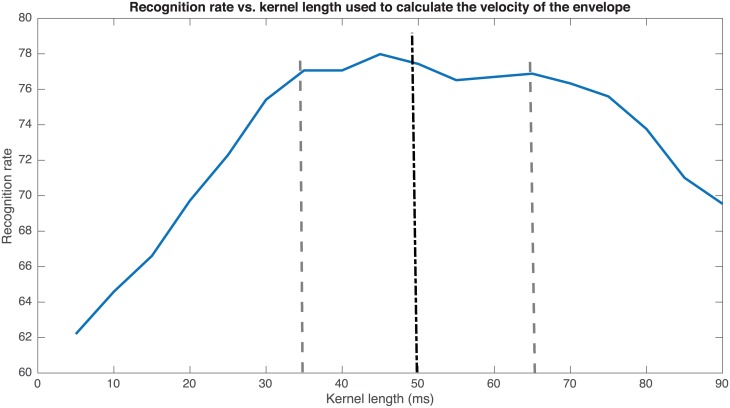
Recognition rate vs. kernel length used to calculate the velocity of the envelope. The optimum is considered to be in the interval [30–65] ms.

**Fig 8:**
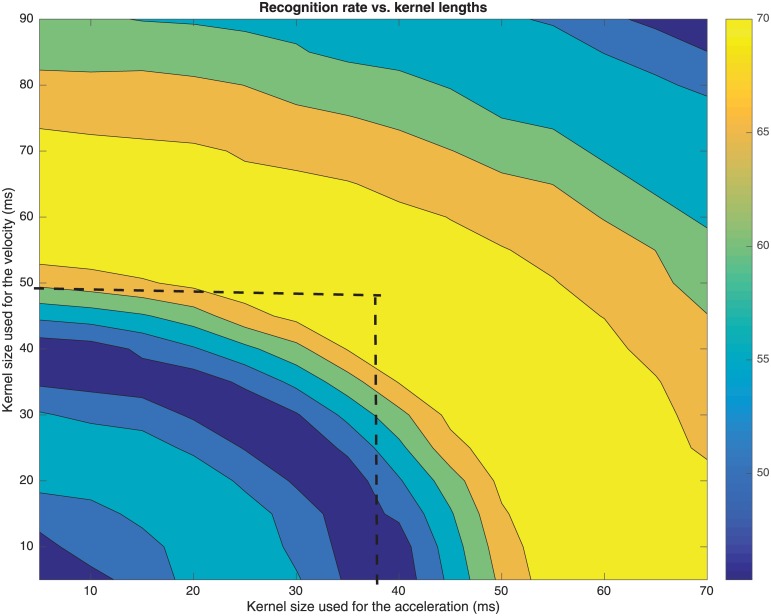
Recognition rate vs. kernel lengths used to calculate the velocity and acceleration of the envelope. A 50 ms long kernel for the velocity corresponds with a 40 ms kernel for the acceleration.

Fig 7 shows a plot of the recognition rate at the EER obtained vs. the length of the kernel used to estimate Δ. The curve shows a plateau between 35 and 65 ms, so its centre (i.e. 50 ms) is considered the most stable point of operation. In addition, Fig 8 shows the recognition rate at the EER obtained vs. the length of the kernels used to respectively estimate Δ and ΔΔ. Best results are obtained in the yellow strip of Fig 8, so having in mind the aforementioned selection of 50 ms for the kernel of the Δ, a good tradeoff is a 40 ms long kernel for the ΔΔ, which is again in consonance with the aforementioned constrain limit of 70–90 ms to avoid an oversmoothing effect. The length of these kernels introduces a smoothing effect that partially removes the short term amplitude perturbations during the phonation of the vowels (i.e. shimmer), but keeps the amplitude tremor, which for PD patients is typically around 3–7 Hz.

**Fig 9:**
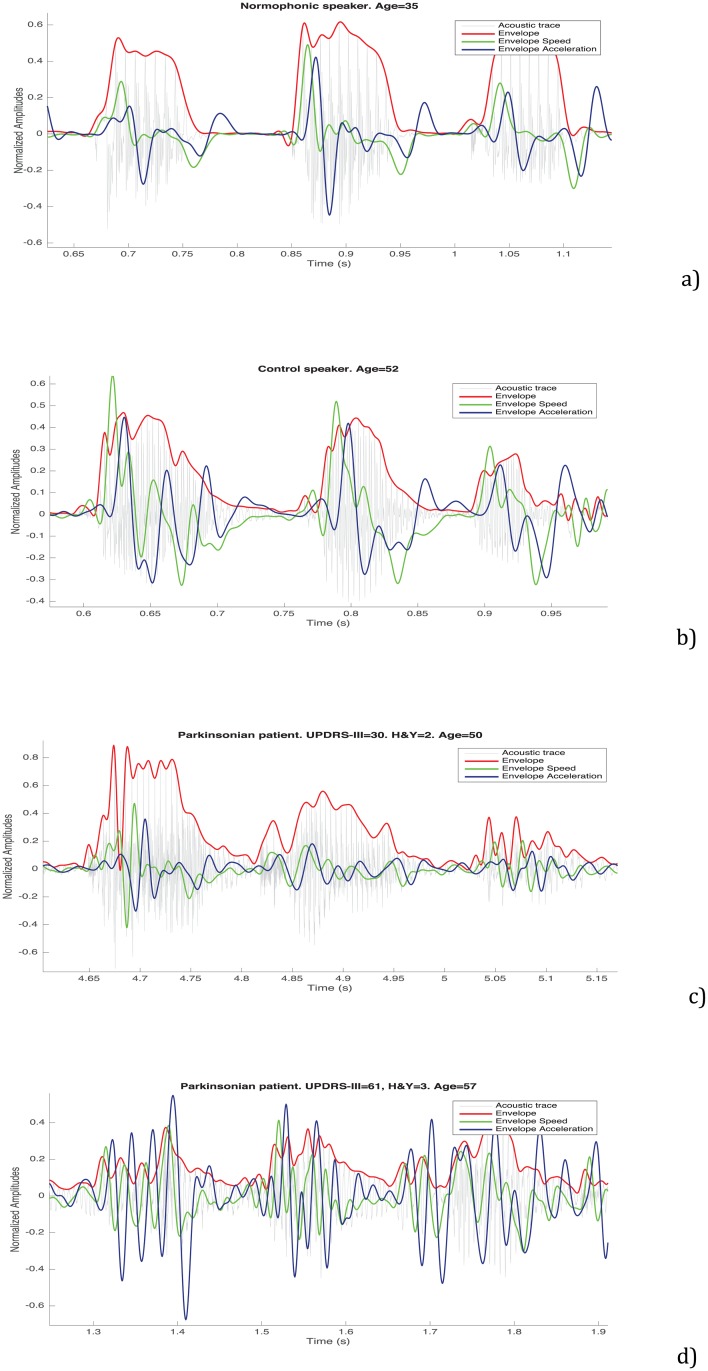
Speech trace with its envelope and an estimate of the velocity and acceleration of the envelope for a young normophonic 35 years old person (a), a control speaker (b), a parkinsonian patient with H&Y = 2 (c), and a parkinsonian patient with H&Y = 3 (d), all of them calculated using 50 and 40 ms. long smoothing kernels for the velocity and acceleration respectively. The speech traces correspond to one single utterance of the /pa/-/ta/-/ka/ test. The amplitudes are normalized in the range [–1, 1] for each 1.37 s long frame of analysis. Note that the time scales are different for each plot due to a different speech rate.

**Fig 10:**
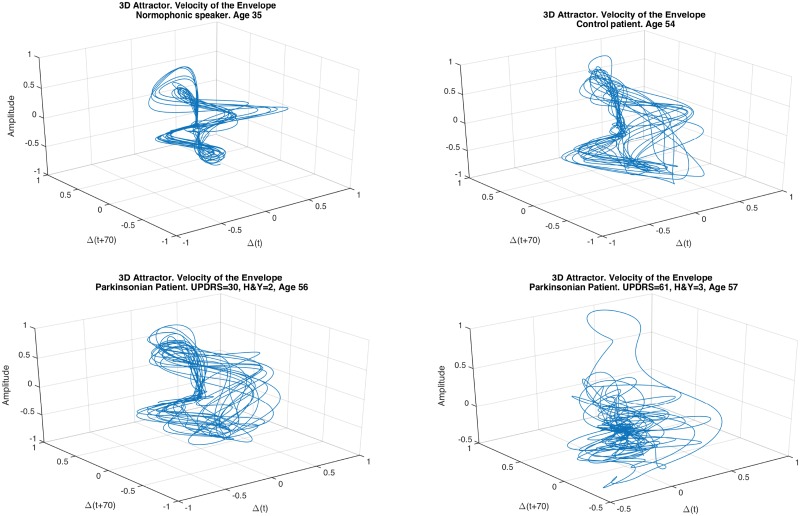
3D attractors of the envelope speed for a young normophonic 35 years old person (a), a control speaker (b), a parkinsonian patient with H&Y = 2 (c), and a parkinsonian patient with H&Y = 3 (d), all of them calculated using 50 ms long smoothing kernel for the speed and a time delay of 70 samples.

Fig 9 shows four prototypical plots corresponding to the envelope, velocity and acceleration sequences of four speakers uttering the /pa/-/ta/-/ka/ test: (a) corresponds to a young normophonic 35 years old person not belonging to the corpus of speakers used; (b) to a control speaker; (c) to a parkinsonian patient with H&Y = 2 and UPDRS-III = 30; and, (d) to a parkinsonian patient with H&Y = 3 and UPDRS-III = 61. All of them were calculated using the durations estimated for the smoothing kernels. A simple analysis of the envelope allows identifying a deterministic trace with a stochastic behaviour superimposed. The deterministic component is due to the alternation of the different syllables, and the stochastic component is due to non desired changes or inaccuracies of the articulators that lead to the distortions described in the previous sections. Ideally speaking, the deterministic component of the envelope should be a sort of rectangular pulses train with short and small peaks during the low amplitude values corresponding to the burst instants. Fig 10 shows the 3D attractors corresponding to the velocity of the envelope for the same speakers represented in Fig 9 for a fixed τ = 70. The trajectories of the attractor are more stable for the normophonic and control speaker.

In the second stage of the methodology, each of the frames corresponding to Δ and ΔΔ have been parameterized by means of a more complete analysis that involves 7 classic complexity measurements: Largest Lyapunov Exponent (LLE), Correlation Dimension (CD), Hurst Exponent (HE), Detrended Fluctuation Analysis (DFE), Recurrence Period Density Estimation (RPDE), Gaussian Kernel AE (GAE), Fuzzy Entropy (FE), and Permutation Entropy (PE). All these measures were extracted using the τ and *D* values calculated following the aforementioned procedures. Fusing the Δ and ΔΔ information led to a feature vector with 14 components. Even when literature reports other complexity measures that could be used, and even with better discrimination capabilities, for the sake of simplicity only the most classical have been used.

These features were finally used to feed a supervised discriminative system based on a support vector machine (SVM) with a linear kernel to binary categorize each frame of speech as normophonic or parkinsonian. Once again, the decision of using one or another classifier and its configuration was guided by a rule of simplicity.

In a first step, and to ensure the generalization capabilities of the classifier, the database is used to train and test several models following a k-folds cross validation procedure using 5 folds. A decision is taken for each frame of analysis, and the final score assigned to each speaker is calculated multiplying all the scores obtained for each frame and normalizing with the number of frames. These preliminary results are used to determine the optimal parameters of the SVM.

Then, a k-folds technique with 11 folds is used in the same database to estimate the overall performance of the system, employing the SVM parameters determined in the previous step.

Once the accuracy of the proposed scheme has been estimated, an algorithm based on Sequential Floating Feature Selection (SFFS) [72] has been applied to reduce the dimensionality of the feature space, as well as to eliminate redundant information. This method finds the best subset of features starting from the original set from which includes and excludes features. In this procedure after each forward step, a number of backward steps are applied in order to identify the resulting subsets providing better accuracy than the previous ones.

## Results

This section presents the results obtained for the baseline system based on speaker verification techniques as well as those obtained using the kinematic approach proposed in this paper.

### *Framework 1*. Speaker verification techniques

**Fig 11:**
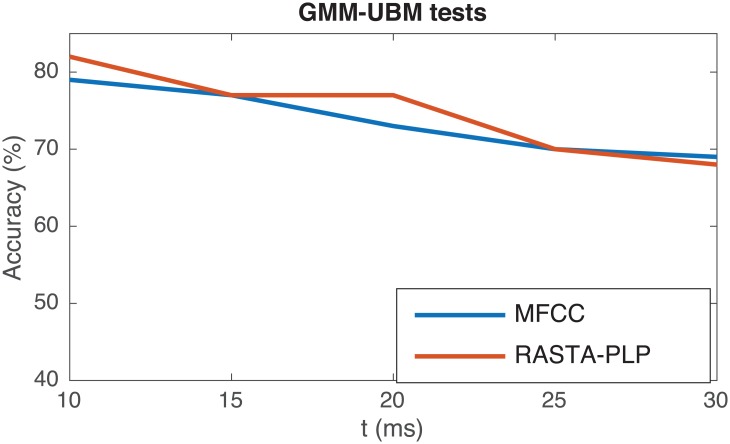
Accuracy vs. window size for a GMM-UBM system trained with 128 gaussians for two different parameterization approaches. Best results are with 10 ms. windows.

In all the performed tests with GMM-UBM schemes, best results are obtained using 10 ms window lengths, independently of the parameterization technique as it can be inferred from Fig 11. The best global results obtained for each combination of parameterization technique and classification scheme are summarized in Table 2 along with the sensitivity and specificity of each test.

**Table 2:** Best results in terms of accuracy, area under the ROC curve, sensitivity and specificity for both configurations (GMM-UBM and iVectors) and parameterization approaches (MFCC and RASTA-PLP).

		Sensitivity	Specificity	AUC	Accuracy
MFCC	GMM-UBM	0.8O	0.78	0.87	79.0±7.9%
	iVectors	0.80	0.78	0.86	79.0±7.9%
RASTA-PLP	GMM-UBM	0.82	0.82	0.86	82.0±7.5%
	iVectors	0.82	0.82	0.90	82.0±7.5%

Regarding the MFCC parameterization modeled using GMM-UBM, best results (AUC = 0.87, 79% of accuracy) were obtained using 64 components for the Gaussian mixture and 18. The iVectors approach delivered the best results (AUC = 0.86, 79% of accuracy) using 10 MFCCs, 128 components for the Gaussian mixtures, iVectors with a dimension of 30 and vector of latent factor of dimension equal to 2.

With respect to the detection approach based on RASTA-PLP and GMM-UBM, best results (AUC = 0.86, 82% of accuracy) were obtained using 16 components for the Gaussian mixture and 20 coefficients. In the same way, the approach based on iVectors revealed the best results (AUC = 0.90, 82% of accuracy) using 12 coefficients, 256 components for the Gaussian mixtures, iVectors with a dimension of the variability space of 50, and vector of latent factor of dimension equal to 18.

**Fig 12:**
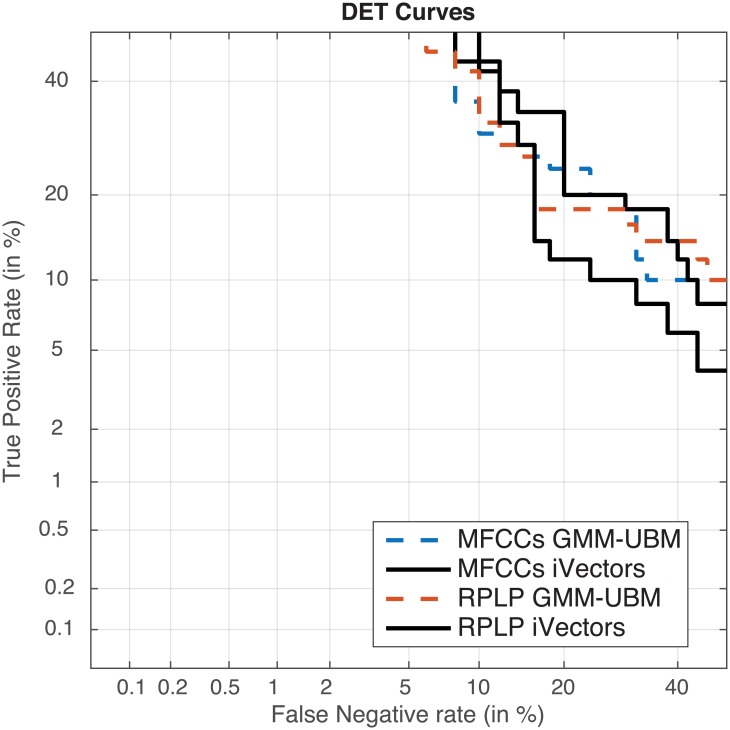
DET curve using GMM-UBM and iVectors approaches for MFCC and RASTA-PLP parameterization approaches.

The accuracy of those configurations that provided the best results is graphically depicted in Fig 12 for both parameterization approaches (i.e. MFCC and RASTA-PLP) and modeling schemes (i.e. GMM-UBM and iVectors) using a Detection Error Tradeoff (DET) plot.

**Fig 13:**
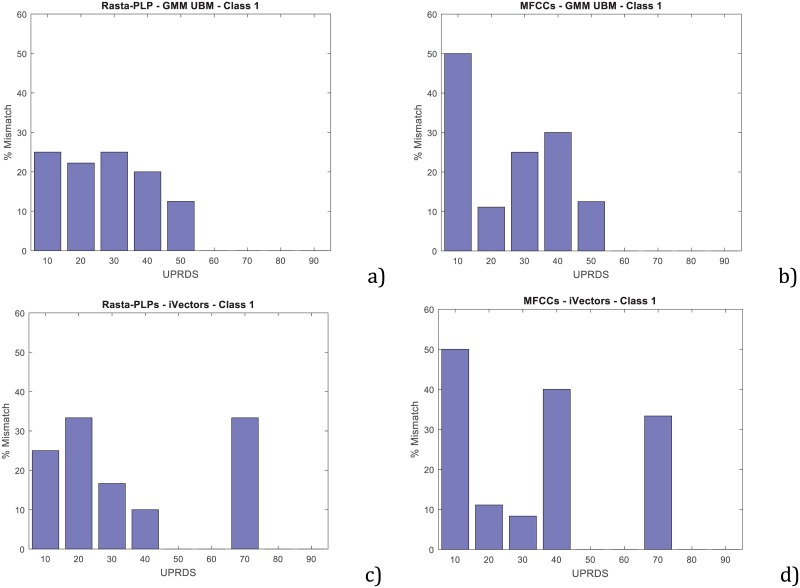
Normalized histograms of the UPDRS-III labels corresponding to the speakers wrongly categorized. a) using GMM-UBM and RASTA-PLP; b) using GMM-UBM and MFCC; c) using iVectors and RASTA-PLP; d) using iVectors and MFCC.

Fig 13 shows the behaviour of the baseline system in the grey zone of decision. The histograms represent the UPDRS-III labels of those PD speakers that were wrongly categorized. Histograms were normalized with the absolute number of speakers in the database for each bin, which are represented in Fig 2. As expected, the lower the UPDRS-III labels of the speaker the more likely the error of the classifier. A similar behaviour appears for the four baseline schemes developed, although there is an undesired behaviour for the iVectors scheme both with RASTA-PLP and MFCC, and UPDRS-III levels of 70 attributable to the small sample size to accurately train the iVectors approach, which usually requires a large amount of data.

### *Framework 2*. Kinetic features

**Fig 14:**
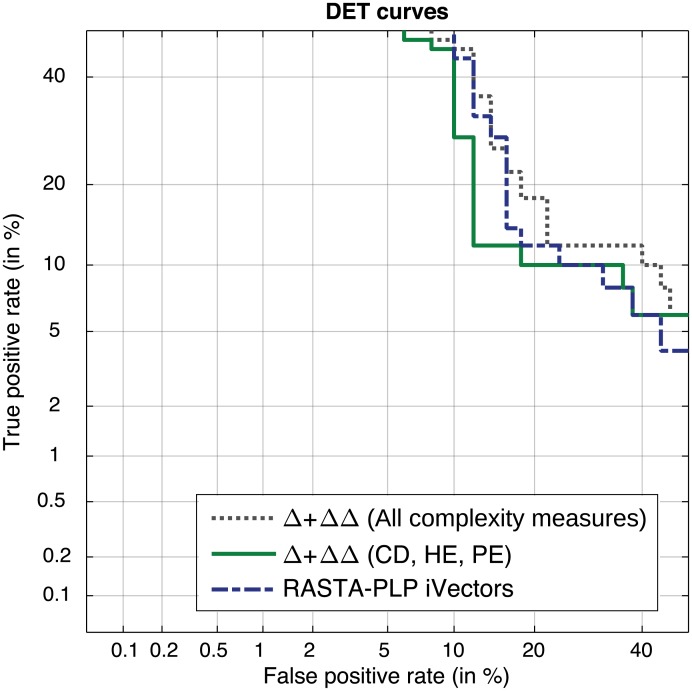
DET plot of the best baseline system and of the proposed method.

Table 3 summarizes the results obtained with the second stage of the proposed method, both in term of frame and file accuracy. The optimal parameters used to calculate velocity and acceleration of envelope are obtained in the preliminary part of the methodology, described in Section 2.6.2. The frame accuracy refers to the number of frames correctly categorized, whereas the file accuracy is linked with the number of speakers adequately categorized. These values include the confidence interval calculated as detailed in [73]. Tests were carried out parameterizing the speed and acceleration of the envelope and fusing their parameters together. Also the results of the best feature set are presented. A combination of the whole set of complexity measures provided and 81.0% of accuracy with an AUC of 0.87, whereas the reduced set reached 85.0% with an AUC of 0.91. Throughout a DET plot, these results are graphically depicted in Fig 14 in comparison with the baseline system inspired in speaker recognition techniques. In both frameworks results are significantly better than the random trial (50%).

**Table 3:** Best results in terms of accuracy ± confidence interval, area under the ROC curve, sensitivity and specificity for both configurations.

		Dim.	Sensitivity		Specificity		AUC		Accuracy	
			Frame	File	Frame	File	Frame	File	Frame	File
All features	Δ	7	0.71	0.76	0.75	0.78	0.79	0.83	76.6±5.0	77.0±8.2
	ΔΔ	7	0.76	0.80	0.76	0.82	0.80	0.87	76.0±4.8	81.0±7.7
	Δ+ΔΔ	14	0.77	0.80	0.77	0.82	0.81	0.87	77.5±4.8	81.0±7.6
CD, HE, PE	Δ	3	0.76	0.76	0.77	0.82	0.82	0.87	76.8±4.8	79.0±7,9
	ΔΔ	3	0.74	0.80	0.73	0.80	0.79	0,88	73.3±5.0	80.0±7.8
	Δ+ΔΔ	6	0.77	0.82	0.79	0.88	0.84	0.91	78.1±4.7	85.0±6.9

**Fig 15:**
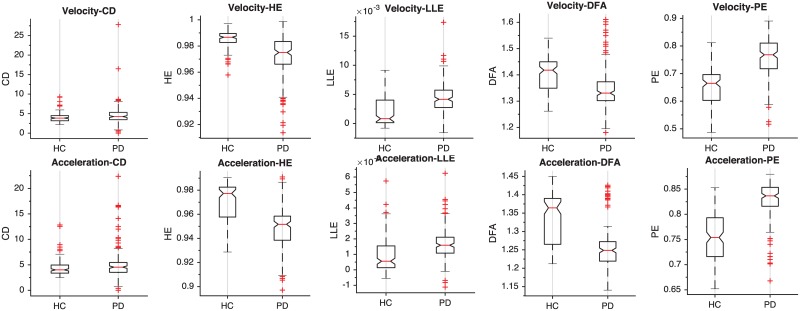
Boxplots corresponding to the complexity measures extracted from the acceleration (top row) and velocity (bottom row) sequences.

Fig 15 shows the boxplots of the complexity features applied to the velocity and acceleration that have demonstrated better discrimination capabilities for the proposed problem. The boxplots clearly show the discrimination capabilities, especially for PE, DFA, and HE. In view of the boxplots, CD does not show strong discrimination capabilities, but in contrast results suggest that contributes with additional information.

**Fig 16:**
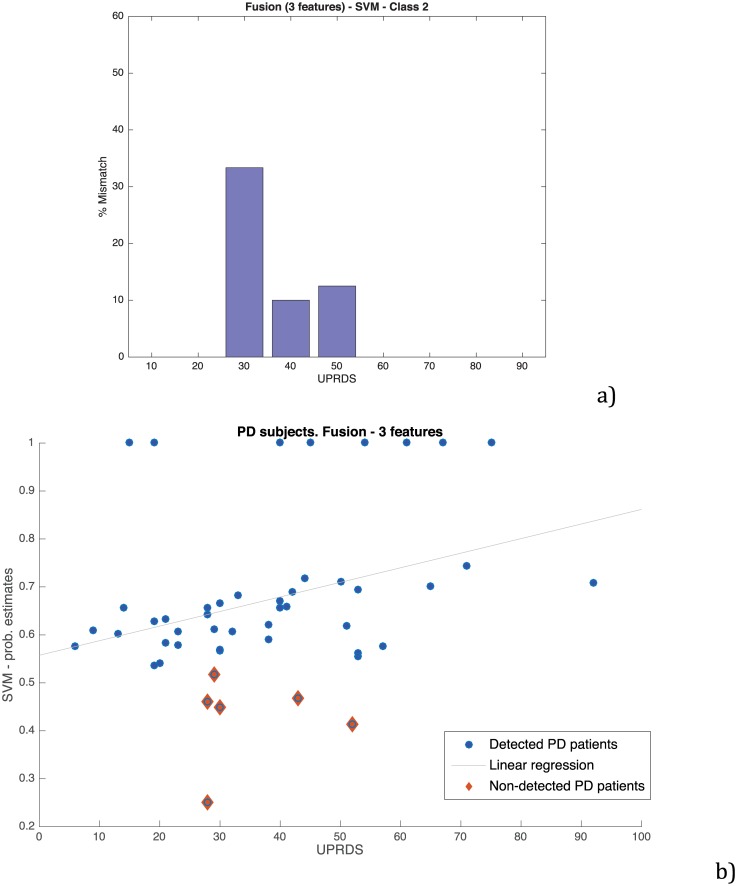
a) Normalized histogram of the UPDRS-III labels corresponding to the speakers wrongly categorized with the proposed method, b) UPDRS-III level vs. score given by the proposed method.

As already done with the baseline system, a study of the behaviour in the gray zone was carried out for the best configuration identified. Fig 16a shows that the pathological speakers that were wrongly categorized are concentrated in the range 30–50 of the UPDRS-III scale. A detailed analysis of them revealed that the signs of hypokinetic dysarthria of these speakers are not very strong, which is in consonance with the statistics that show that only 70–90% of the PD patients develop hypokinetic dysarthria during the course of the disease. Finally. Fig 16b shows a regression plot of the score given by the system and the UPDRS-III scale of the PD patients of the database. Dots in orange are those PD patients that were wrongly categorized as normal. With the exception of the dots that appear at the top of the figure, a correlation is observed between the scores and the UPDRS-III labels assigned to each speaker.

## Discussion & conclusions

The DDK test used throughout this paper involves a rapid alternation of the articulators between voiceless stops and vowels. However, the analysis of the examples shown demonstrates a clear tendency of the voiceless obstruents towards the expected pattern of voiced stops. The literature has demonstrated that voiceless obstruents are "fortis," i.e. involve greater muscular force, and voiced obstruents are "lenis". This is in consonance with the second hypothesis of this work, which stands that the speech of PD patients shows a lowering of the effort placed at the level of the articulators. On the other hand, the passive voicing observed during the stops reveals that the air flow is not completely interrupted during the obstruents of the DDK test, meaning that the envelope, and thus its velocity, tends to follow a smoother pattern, with less abrupt changes. This is in consonance with the first hypothesis of this work.

On the other hand, the tremor at phonation level can be interpreted as a small amplitude modulation both in the effort needed to sustain the phonation, and of the air flow that passes through the glottis. Therefore, under the assumption that the envelope of the speed is directly related with the intensity, that the intensity is directly related with the air flow, and that the extent of the mid-term variations of the intensity are directly related with the forces at the articulators level, changes in the acceleration and velocities of the envelope are supposed to be directly linked with the disease.

Additionally, the examples presented in this paper suggest for each syllable clear influences of the previous vocalic phoneme, suggesting an inertial behaviour of the motoric system. Thus, in PD, there seems to be a difficulty in restoring a more rapid selection of the cues for the next stage of motor activation, resulting in a certain tendency to shorten the stop gaps and voice onset times, to lengthen the vowel onset and offset times, and to decrease the amplitude of the stable part of the vowels (i.e. the slope is shallowed in the vowel onset and specially in the offset). This is reflected in the waveform of the DDK test, and indirectly in the velocity and acceleration of its envelope. Moreover, the shimmer and amplitude tremor present during the stable part of the vowels are also present in the envelope, so their changes in terms of velocity and acceleration are also characterized.

A special attention requires the study of the influence of the speech rate in the results obtained. As commented in the introduction, the literature reports that the syllable rates of the DDK test are modified in PD patients due to the difficulties to rapidly alternate speech movements [54]. However, the complexity measures extracted from the speed and acceleration of the envelope are supposed to be independent of the speech rate, due to the adaptive estimation of the time lag used to reconstruct the attractor. This means that the parameters obtained are not expected to be biased by deviations of the syllable rate, being complementary to this measure.

With respect to the frame length used to analyse the velocity and acceleration sequences, the larger the duration of the frame the better the posterior estimation of the parameters would be. However this value was fixed to 1.37 s for practical purposes, since this is the maximum duration of one of the recordings available in the database. This is a limitation of the corpus used, therefore its impact should be verified with a more complete database of speakers.

The rationales that motivated the design of the proposed biomarkers are simplicity and easiness of interpretation, as well as non-complex parameterization and classification procedures. Besides, these have been the motivations to select the different degrees of freedom of the algorithm, as well as to identify the feature set and the machine learning techniques used. Even when the final figure of merit is the accuracy to categorize control and parkinsonian speakers, maximizing it is not the main criterion that has guided the goals of the present research. The main aim has been the identification of a new set of articulatory features that would demonstrate a strong correlation with the disease. On the other hand, the acoustic biomarkers developed in this work are not presented with the aim to be used alone for the identification of PD. A more holistic approach would require a multidimensional analysis in conjunction with other features at phonetic, articulatory and prosodic levels.

Although with certain differences, results suggest that the frameworks compared in this work provide analogous accuracies. Even when the proposed approach seems to give better results than the baseline system used for comparison purposes (85% vs. 81%), the size of the database, the confidence intervals of the accuracy, and a sense of prudence let us conclude that both frameworks perform in a similar way. However the proposed approach is preferred for its simplicity and its physical interpretation, as well as the easiness to tune its different degrees of freedom. According to the needs expressed by clinicians, these are crucial aspects that must guide the design of new systems in order to be transferred to a clinical setting.

With respect to the baseline system, the first and second derivatives of the MFCC and RASTA-PLP parameters were used because they are supposed to carry important information about the variability of the articulators during the speech, which is a crucial aspect to analyse the speech of PD patients. Besides, it is important to note that the time length of the kernel used to estimate these derivatives has been kept constant with independence of the window size, thus ensuring that the smoothing effect is the same for all the window lengths considered. To the best of our knowledge this aspect has never been addressed before in the literature and requires a special attention and a further study.

Despite of light differences in terms of accuracy with respect to MFCC, RASTA-PLP parameters are preferred for an application like the one presented in this paper, since they have a closer physical interpretation. Regarding this family of parameters, RASTA filtering is considered of special support to the PLP parameters, since it eliminates the influence of the spectral components that change more quickly or slowly than the usual rate of change of speech. Therefore, RASTA would enhance effectively transitions between different speech segments.

With respect to the modelling, GMM-UBM provided results comparable to the iVectors approach. The second method has demonstrated better modelling characteristics in applications of speaker verification and recognition with large corpora, but the variability of the dataset used in this work is not enough to take advantage of the benefits introduced by this technique. This assumption is also supported by the small dimension of the total variability space, which is similar to the dimension of the original feature space. Moreover, the analysis of the errors committed by the GMM-UBM system reveals that, as expected, they are more concentrated in speakers belonging to a lower UPDRS-III category.

As commented in the introduction, statistics show that approximately 75–90% of patients with PD develop dysarthria during the course of the disease [4][9][7][8]. It means that this is probably the ceiling expected for the sensitivity of a potential system of these characteristics. The accuracy of the proposed system is in line with the aforementioned statistics. However, the literature has reported speech-based methods of detection that present accuracies beyond this barrier. But a close look to the methods proposed in some of these works let us be cautious when interpreting their results. As a matter of example, [74] reported accuracies over 96% with the same corpus than the one used in the present study. Among other schemes, the authors also used a GMM-UBM approach to automatically detect parkinsonian speech using the DDK test, thus the comparison of the results is straightforward. However the authors of this work trained an UBM with all the speakers of the same database used to adapt the models and to evaluate the performance of the system. This fact would bias the results towards a more optimistic accuracy. Moreover, the window size used in that work is of 40 ms, which violates the quasy-stationary assumption commonly used in speech technology. Note that the DDK test is a mixture of periodic, aperiodic and stochastic signals, hence it is non-stationary by nature, and a good tradeoff, commonly used in speech technology, is to assume that that speech is stationary over a short time interval (around 10–30 ms).

On the other hand, the work in [12] reviewed up to nine different works, all of them evaluated using the corpus developed in [75]. Accuracies ranging from 91 to 99% are reported in this study. But a close look to the methodology followed reveals some common aspects that suggest that the results are overestimated. The corpus, corresponding to recordings of sustained vowels, contains several sessions per speaker, and crossvalidation was carried out at recording session level instead of at speaker level, thus all patients were part of the training and validation sets. Therefore speaker independence is not guaranteed in the experiments, which again would lead to an overestimation of the results by modelling the speaker rather than the presence of the disorder.

In terms of performance, from the clinical point of view, results are more realistic in [76], but the authors used very high dimensional feature vectors (several thousands) with a database of less than 180 speakers. The best results reported in the cited study are of 86.5%, evaluating only read sentences, but due to the feature space dimension is unbalanced with respect to the number of speakers, there is a certain risk of overfitting to the corpus used. Also [77] reported results using a very high dimensional feature space.

The work in [78] considered a small group with 20 early PD patients and 15 controls, reporting categorization accuracies around 80% using different articulation measures. [79] used a dataset of only 46 speakers, 24 of them with PD, to model articulatory deficits from a DDK test, reporting classification results around 88%.

Although comparisons are not straightforward in some cases due to the use of different methodologies and corpora, in view of the state of the art, and in terms of accuracy, we can conclude that the results presented in this work are comparable to others found in the state of the art. But the main advantage of the proposed method is its simplicity and easy physical interpretation.

Throughout this work, we have used the UPDRS-III scale as a ground truth to evaluate the extent of the disease of PD patients. But this aspect deserves a deeper attention and analysis. The UPDRS-III scale has been employed in many of the works already published in the literature, since it is widely used in the clinic and its usefulness is undoubtable. However, it was not specifically designed to evaluate the speech, but also more generic motoric limitations, and only takes into account the effect in the speech in advanced stages of the disease, and with a small weight in the total score proposed by the scale. Thus, its use as a ground truth to evaluate the speech is limited, and conclusions have to be extracted having in mind these concerns. Other perceptual scales, specifically developed to evaluate the speech, such as GRBAS, or specifically developed to evaluate dysarthria, such as Roberston Dysarthria Profile (RDP) [7], or Frenchay Dysarthria Assessment [80], should be taken into account. A validation procedure in PD patients showed that the RDP has a reasonable reliability, absence of ceiling and floor effects, sufficient internal consistency and scaling assumptions, and considerable correlations with UPDRS-III. The authors in [7] also concluded that speech and voice disturbances are well identified by the RDP in early PD patients, even when these deviations do not produce a notable level of disability. As in UPDRS-III, similar considerations can be done with respect to the H&Y scale, but having in mind that this scale does not consider the speech in any case.

Despite of the promising results already published in the literature, and those reported in this paper, it is worth to note that these biomarkers only represent the first step in the automatic screening of PD from the speech. A further study would require a comparison with other types of dysarthrias to analyse the specificity of the test, since different types of dysarthria share some common characteristics.

## Appendix

This appendix describes the procedures used to estimate the different parameters that have to be adjusted prior to the construction of the embedded attractors.

**Fig 17:**
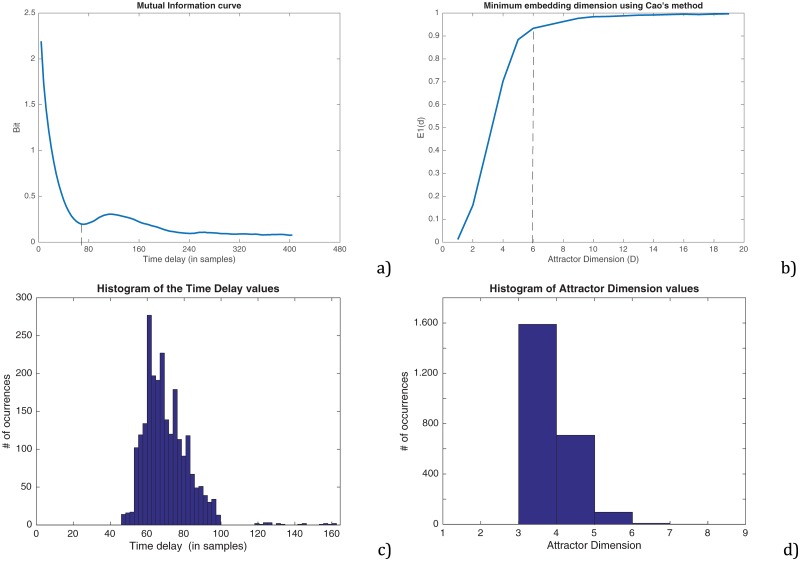
Example of the estimation of the time lag (a) and embedding dimension (b) for a 1.37 s. long frame corresponding to the velocity of variation of the envelope of a normophonic speaker during the /pa/-/ta/-/ka/ test. In this example, the first minimum of the auto mutual information can be found at 70. Regarding the embedding dimension, the plot of the E1 value used for the Cao’s method shows a kink at 6. The histograms in (c) and (d) correspond to the time delays and embedding dimensions respectively obtained for all the frames extracted from the database.

The estimation of the embedded atractors has two degrees of freedom: the time lag and the embedding dimension. Adjusting these parameters is important to ensure that the entropy values are measuring the complexity of the signal with independence of the dynamics of the deterministic part of the speech (e.g. variations due to the speech rate). The time lag, τ, is typically fixed to 1 but a better estimation might give information associated to the intrinsic time scales of the system. In this case, τ was calculated for each 1.37 s frame using a criterion based on the estimation of the first minimum of the auto mutual information [81] (Fig 17a). In contrast to other criteria also used in the literature, such as the first zero crossing of the autocorrelation function, the first minimum of the auto mutual information is not connected to linear or nonlinear evolution rules of the signal. It connects two sets of measurements and establishes a criterion for their mutual dependence based on the notion of information connection between them.

On the other hand, the embedding dimension, *D*, is estimated based on the Cao’s method [82], which is run using four nearest neighbours, a maximum dimension of 15, and the τ value calculated in the previous step (Fig 17b). To achieve a reliable statistics and proper discrimination between stochastic and deterministic dynamics, it is necessary that N>>D!, being N the length of the segment (in samples) to analyse. For the sake of simplicity, and considering 3 ≤ D ≤ 7, a constant *D* = 6 value was estimated for the whole dataset, taking the maximum value of the individual embedding dimensions obtained for each of the speech frames (Fig 17d). No significant differences in the final accuracy of the system were found with respect to the use of a fixed or an adaptive *D* value estimated for each frame of analysis.

Typical τ values are in the range {45, …, 100} samples, which in combination with *D* = 6, means that, roughly speaking, each point in the embedded space contains information of a temporal environment that is equivalent to (*D* − 1)· τ/*f*_*s*_ = {45, …, 100} ms. (being *f*_*s*_ the sampling frequency of the sequences, fixed to 5 kHz). The largest τ values are expected to correspond to those patients with a lower rate of speech and vice-versa.
